# ERCC1 and TS Expression as Prognostic and Predictive Biomarkers in Metastatic Colon Cancer

**DOI:** 10.1371/journal.pone.0126898

**Published:** 2015-06-17

**Authors:** Michel B. Choueiri, John Paul Shen, Andrew M. Gross, Justin K. Huang, Trey Ideker, Paul Fanta

**Affiliations:** 1 Department of Medicine, University of California San Diego, La Jolla, California, United States of America; 2 Bioinformatics and Systems Biology Program, University of California San Diego, La Jolla, California, United States of America; 3 Moores Cancer Center, University of California San Diego, La Jolla, California, United States of America; Queen Mary Hospital, HONG KONG

## Abstract

In patients with metastatic colon cancer, response to first line chemotherapy is a strong predictor of overall survival (OS). Currently, oncologists lack diagnostic tests to determine which chemotherapy regimen offers the greatest chance for response in an individual patient. Here we present the results of gene expression analysis for two genes, ERCC1 and TS, measured with the commercially available ResponseDX: Colon assay (Response Genetics, Los Angeles, CA) in 41 patients with de novo metastatic colon cancer diagnosed between July 2008 and August 2013 at the University of California, San Diego. In addition ERCC1 and TS expression levels as determined by RNAseq and survival data for patients in TCGA were downloaded from the TCGA data portal. We found that patients with low expression of ERCC1 (n = 33) had significantly longer median OS (36.0 vs. 10.1 mo, HR 0.29, 95% CI .095 to .84, log-rank p = 9.0x10^-6^) and median time to treatment to failure (TTF) following first line chemotherapy (14.1 vs. 2.4 mo, HR 0.17, 95% CI 0.048 to 0.58, log-rank p = 5.3x10^-4^) relative to those with high expression (n = 4). After accounting for the covariates age, sex, tumor grade and ECOG performance status in a Cox proportional hazard model the association of low ERCC1 with longer OS (HR 0.18, 95% CI 0.14 to 0.26, p = 0.0448) and TTF (HR 0.16, 95% CI 0.14 to 0.21, p = 0.0053) remained significant. Patients with low TS expression (n = 29) had significantly longer median OS (36.0 vs. 14.8 mo, HR 0.25, 95% CI 0.074 to 0.82, log-rank p = 0.022) relative to those with high expression (n = 12). The combined low expression of ERCC1/TS was predictive of response in patients treated with FOLFOX (40% vs. 91%, RR 2.3, Fisher’s exact test p = 0.03, n = 27), but not with FOLFIRI (71% vs. 71%, RR 1.0, Fisher’s exact test p = 1, n = 14). Overall, these findings suggest that measurement of ERCC1 and TS expression has potential clinical utility in managing patients with metastatic colorectal cancer.

## Introduction

Colorectal cancer remains the third deadliest cancer in the United States with an estimated incidence of 132,700 new cases and 49,700 deaths in 2015. The introduction of new cytotoxic and targeted agents for patients with metastatic CRC (mCRC) has improved overall survival (OS) rates, with expected median survival now in excess of 20 months with many patients surviving beyond two years[1]. The current treatment paradigm consists of 5-Fluorouracil based regimens in combination with either oxaliplatin (FOLFOX) or irinotecan (FOLFIRI), potentially combined with therapy targeting either EGFR or VEGFR [2]. Prior large, prospective clinical trials have shown that when used as first line therapy options, response rates (RR) for either FOLFOX or FOLFIRI are around 55% [3]. Currently, oncologists have limited diagnostic tools to predict which first line chemotherapy option is best for an individual patient. Choice of first line therapy is of great importance in mCRC as it has been shown that patients who respond to first line therapy have longer OS[4], and some of these patients with oligometastatic disease can proceed to undergo metastatectomy with curative intent[5].

Many potential predictive biomarkers for mCRC have been reported using a variety of molecular data types [6–8]. These markers include the mutation status of KRAS[9, 10], BRAF [11, 12] and TP53[13–15]. Other prognostic markers include microsatellite instability (MSI)[16, 17], epidermal growth factor receptor (EGFR) copy number[18], 18q allelic imbalance, TP53 expression as well as the expression levels of Thymidylate Synthase (TS) [13, 19] and Excision Repair Cross-Complementation Group 1 (ERCC1) [6, 20]. However, outside the exception of KRAS mutation status predicting response to the anti-EGFR antibodies cetuximab and panitumumab, the demonstrated clinical value of other biomarkers has been limited [21, 22].

ERCC1 and TS expression levels have been previously described as potentially promising biomarkers in mCRC [23]. ERCC1 is known to be involved in the nucleotide excision and repair pathway, a part of the cellular response to DNA damage. Patients with low levels of ERCC1 expression have been reported to have an improved response and a longer OS in gastrointestinal tumors treated with FOLFOX [23–26]. TS is an enzyme responsible for the generation of deoxythymidine triphosphate (dTMP), which is needed for the formation of the nucleic acid thymine. Most notably, TS is the target of the commonly used antimetabolite 5-Fluorouracil (5-FU). TS gene expression has been shown to be predictive of response to 5-FU-based therapy in patients with mCRC [27–30]. Low levels of TS have also been correlated with improved response rate and OS in patients treated with FOLFOX, but had no prognostic value in patients treated with FOLFIRI [23, 27, 30]. Because gene expression is a continuous variable, for diagnostic purposes it is important to establish a threshold level that defines a population with high expression and a distinct population with low expression. For ERCC1, threshold values have been suggested based on response to platinum-based chemotherapy in colon, gastric, head/neck, bladder and non-small-cell lung cancer [23]. Specific to mCRC, the CONFIRM-1 and -2 trials included 122 patients with mCRC and established the clinical utility of existing thresholds for high vs. low ERCC1 and TS gene expression as measured by qPCR and normalized against actin[6]. In those studies, patients with high TS and/or high ERCC1 showed shorter OS of 15.6 months compared to 37 months in patients who had low expression levels of both genes.

In view of these data, we have incorporated at our institution routine measurement of ERCC1 and TS expression as part of standard care for mCRC patients. Here we review our single institution cohort of patients with de novo mCRC as well as a cohort from The Cancer Genome Atlas (TCGA) to assess the clinical utility of ERCC1 and/or TS expression gene expression levels.

## Materials and Methods

### Study Design

For this study, we performed a retrospective analysis on two cohorts of patients with known ERCC1 and TS expression levels. Treatment time to failure (TTF) to first line chemotherapy was chosen as primary end point. Secondary end points included objective response rate (ORR) to therapy and OS. TTF was calculated from the start of treatment with either FOLFOX or FOLFIRI until clinical or radiologic evidence of progression. OS was calculated from the time of initiation of treatment until death from any cause. ORR was calculated as the sum of complete or partial responses, determined by improvement in either serial imaging or serial biomarker measurement. Eligibility criteria were defined as follows: Pathological diagnosis of mCRC, first line of treatment with either FOLFOX-6 or FOLFIRI, imaging with computed tomography (CT) within at most three months of the start of therapy. The exclusion criteria included prior adjuvant chemotherapy, advanced comorbidities defined as a recent myocardial infarction or unstable angina, uncontrolled hypertension or diabetes mellitus, hepatic or renal failure. Patients with incomplete or unavailable clinical records were also excluded. Patients treated with concurrent bevacizumab or cetuximab in addition to cytotoxic chemotherapy were included in the analysis. The UCSD Institutional Review Board approved this study. Given its retrospective nature it represented no more than minimal risk to the subjects, therefore per the Code of Federal Regulations on the Protection of Human Subjects a waiver of informed consent was granted. Data for TCGA cohort were obtained from the Genome Data Analysis Center (GDAC) Firehose website. All data were downloaded from the 15 January 2014 standard data and analyses run.

### Molecular Analysis

The ResponseDX: Colon assay includes quantitative PCR to measure gene expression for ERCC1, TS, and VEGFR as well as specific mutations. Extracted DNA was used to detect mutations in exon 2 of KRAS using PCR with primers specific to known mutations[18]. The threshold values for the normalized gene expression measurements used to stratify patients into high or low expression groups were 4.0 and 1.73 for TS and ERCC1, respectively. These threshold values have been previously established and used in the CONFIRM-1 and CONFIRM-2 trials[6]. Kaplan–Meier functions were used to estimate the survival probabilities. Gene expression measurement for TCGA patients was performed using RNAseq, as described[31]. Because there was no prior established threshold for ERCC1 or TS levels measured by RNAseq, for TCGA cohort the mean value of ERCC1 and TS was used as threshold.

### Statistical Analysis

Analysis of TTF and OS curves were performed using the Kaplan-Meier method and reported as hazard ratio (HR), significance of differences between groups was assessed using the log-rank method. Differences in the distribution of categorical variables were assessed using Pearson’s X^2^ test. Differences in ORR are reported as relative risk (RR), significance was tested using 2-sided Fisher’s Exact test. Cox multiple regression analysis of TTF and OS was used to assess the contribution of age, sex, tumor grade, and ECOG performance status. The correlation between ERCC1 and TS was calculated using two-tailed Pearson R test. Statistical calculation was performed using GraphPad Prism version 6.04 and OriginPro 2015.

## Results

### Clinical and Molecular Characteristics

Clinical records of patients with de novo mCRC treated at our center between July 2008 and August 2013 were reviewed. Fifty patients who had their tumors tested by ResponseDX: Colon (Response Genetics, Los Angeles, CA) as part of routine clinical care were identified, 41 met eligibility criteria and were included in the final analysis **(Table 1)**. Complete patient characteristics are summarized in S1 Table. Eighteen patients were male (44%) and 23 were female (56%). The median age was 57.6 years with range from 31 to 86. Twenty-four (59%) of the patients underwent surgery for the primary tumor and 12 (29%) underwent a metastatectomy during the course of their treatment. The majority of patients were treated with first line FOLFOX (66%), receiving an average of 9.5 doses with range 2–20. Approximately one third of patients were treated with first line FOLFIRI (34%), receiving an average of 8.5 doses with range 2–14. Before the initiation of therapy, 93% of patients had an ECOG performance status of 0 or 1. All patients were assessed for ERCC1 and TS expression and KRAS mutation status. Using pre-established cutoff values, 37 patients (90%) had low ERCC1 expression levels and 29 patients (71%) had low TS expression levels. Median expression ERCC1 for and TS was 1.13 and 2.65 respectively. Twenty-two patients (54%) had mutations in KRAS.

**Table 1:** Patient Characteristics.

Characteristic	number of patients (percentage)
Male	18 (43%)
Female	23 (57%)
Well differentiated	1 (2%)
Moderately differentiated	32 (78%)
Poorly differentiated	8 (20%)
FOLFOX (1st line)	27 (66%)
FOLFIRI (1st line)	14 (34%)
ECOG 0	17 (42%)
ECOG 1	21 (51%)
ECOG 2	3 (7%)
Primary resection	24 (59%)
Metastatectomy	12 (29%)

### TCGA cohort

**Fig 1:**
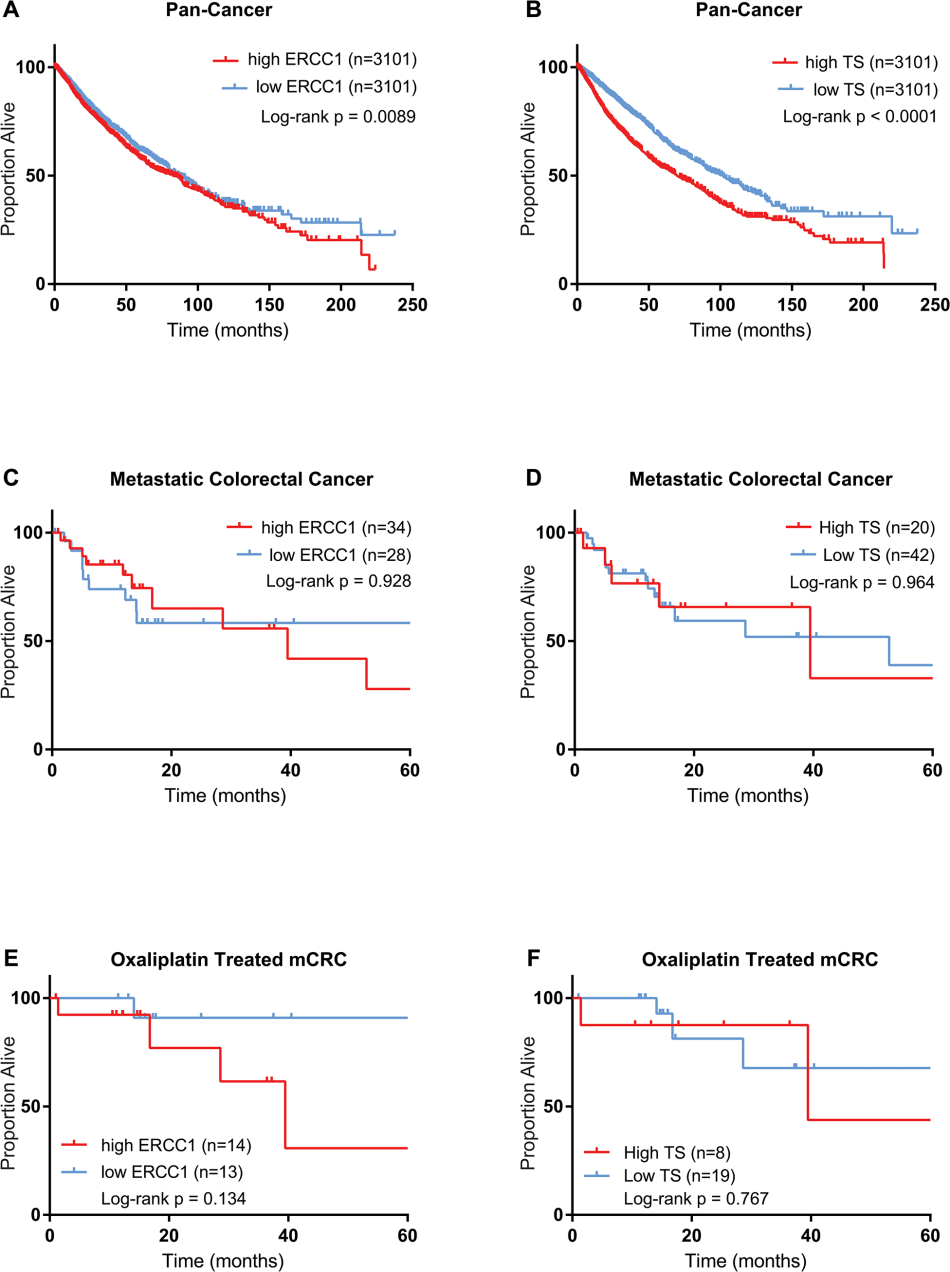
Prognostic value of ERCC1 and TS in TCGA cohort. (A) OS of all patients in TCGA stratified by ERCC1 expression. (B) OS of all patients in TCGA stratified by TS expression. (C) OS of all mCRC patients from TCGA stratified by ERCC1 expression. (D) OS of all mCRC patients from TCGA stratified by TS expression. (E) OS of only those mCRC patients treated oxaliplatin stratified by ERCC1 expression. (F) OS of only those mCRC patients treated oxaliplatin stratified by TS expression.

Clinical and molecular data for a second cohort of patients was obtained from TCGA. In total, 6202 patients in a pan-cancer cohort, including 62 patients with mCRC had data for both OS and gene expression. TTF and response rate were not known for all patients in the TCGA cohort so analysis was restricted to OS. In the pan-cancer cohort of all 6202 TCGA patients, there was minimal difference in median OS between patients with low and high levels of ERCC1 (87.2 vs. 82.9 mo, HR 0.95, 95% confidence interval [CI]: 0.86 to 1.05, p = 0.009) **(Fig 1A).** Patients with low TS levels had a longer median OS compared to patients with high TS levels (102 vs 69.8 mo, HR 0.61, 95% CI: 0.55 to 0.68, log-rank p < 10^−9^) **(Fig 1B).** The effect of ERCC1 was magnified by restricting the patient cohort to only those 1835 patients who received adjuvant chemotherapy (79.3 mo median OS for low ERCC1 vs. 54.9 for high ERCC1, HR 0.72, 95% CI: 0.61 to 0.85, p = 0.0001). In the cohort of all 62 mCRC patients from TCGA, the effect of ERCC1 expression on median OS was not significant (not-yet-reached in patients with low ERCC1 vs. 39.5 mo in patients with high ERCC1, HR 0.96, 95% CI: 0.39 to 2.4, p = 0.93), with hazard ratio similar to that of the pan-cancer cohort **(Fig 1C).** Similarly there was no significant association of TS expression with OS in the full TCGA mCRC cohort (HR 0.98, 95% CI: 0.35 to 2.7, p = 0.96) **(Fig 1D).** Among the 27 mCRC patients treated with oxaliplatin, the hazard ratio for low ERCC1 was one quarter that of the full mCRC cohort; patients with low ERCC1 had median OS not-yet-reached vs. 39.5 months for those with high ERCC1 (HR 0.22, 95% CI: 0.045 to 1.5, p = 0.13) **(Fig 1E).** There was no survival difference between patients with low TS and high TS expression in this population (HR 0.77, 95% CI: 0.12 to 4.8, p = 0.77) **(Fig 1F).**

### UCSD Cohort, ERCC1

**Fig 2:**
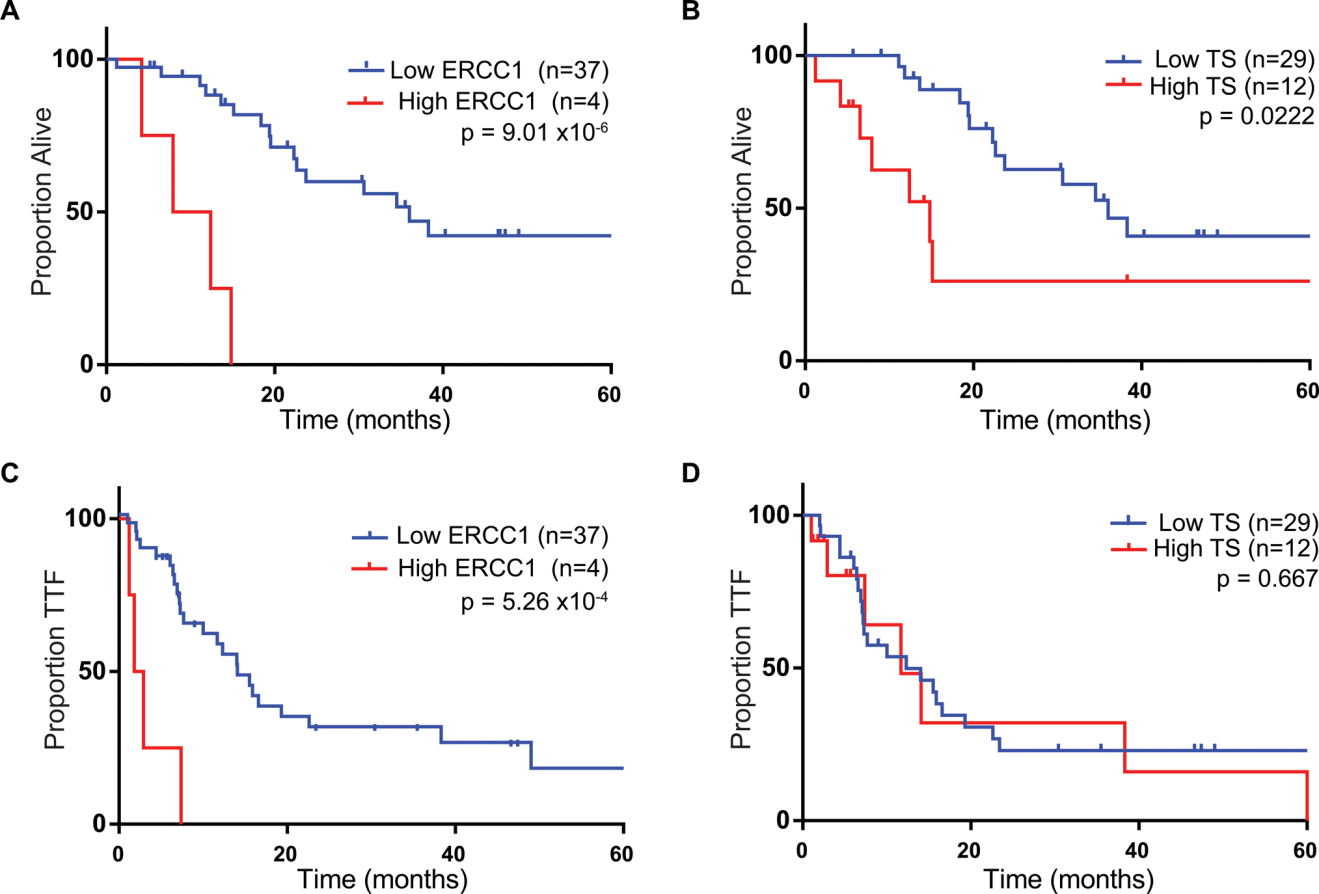
Overall survival and time to treatment failure by gene. (A) OS in all patients stratified by ERCC1 expression. (B) TTF in all patients stratified by ERCC1 expression. (C) OS in all patients stratified by TS expression. (D) TTF in all patients stratified by TS expression.

Thirty-seven patients had ERCC1 expression levels below the cutoff of 1.73; only 4 patients had ERCC1 levels above this threshold. Patients with low ERCC1 had a trend towards higher ORR to any chemotherapy than those with high ERCC1 (83% vs 50%, RR 0.60, 95% CI: 0.22 to 1.6, Fisher’s exact p = 0.16) **(Table 2).** Median OS was significantly longer for patients with low ERCC1 (36.0 vs. 10.1 mo, HR 0.29, 95% CI: 0.095 to 0.84, log-rank p = 9.0x10^-6^) **(Fig 2A).** Median TTF was also longer for patients with low ERCC1 (14.1 vs. 2.4 mo, HR 0.17, 95% CI 0.048 to 0.58, log rank p = 5.3x10^-4^) **(Fig 2B).** After accounting for the covariates age, sex, tumor grade and ECOG performance status in a Cox proportional hazard model the association of low ERCC1 with longer OS (HR 0.18, 95% CI 0.14 to 0.26, p = 0.0448) and TTF (HR 0.16, 95% CI 0.14 to 0.21, p = 0.0053) remained significant. Among the 37 patients with low ERCC1, 24 received treatment with FOLFOX and 13 received FOLFIRI. Those treated with FOLFOX showed a trend towards longer median OS relative to FOLFIRI (36.0 vs. 22.6 months, HR 0.91, 95% CI: 0.32 to 2.4, p = 0.83) but the difference did not reach statistical significance. In patients with low ERCC1, the ORR to FOLFOX was 86% compared to 72% for FOLFIRI (RR 1.3, 95% CI: 0.78 to 2.1, Fisher’s exact p = 0.25).

**Table 2:** Objective response rates per biomarker level.

Group	Objective response rate	P-value (Fisher’ exact test)
Low ERCC1	31/37 (84%)	0.165
High ERCC1	2/4 (50%)	
Low TS	25/29 (86%)	0.092
High TS	7/12 (58%)	
Low ERCC1/ low TS	25/29 (86%)	—
Low ERCC1/ high TS	5/8 (63%)	0.156 (vs. low ERCC1 / low TS)
High ERCC1/ high TS	2/4 (50%)	0.142 (vs. low ERCC1 / low TS)
FOLFOX	22/27 (81%)	0.462
FOLFIRI	10/14 (71%)	

### UCSD Cohort, TS

Twenty-nine patients had TS expression less than the threshold value 4.0; twelve had expression levels above this threshold. The median OS was significantly longer for patients with low TS expression (36.0 mo vs 14.8 mo, HR 0.37, 95% CI: 0.076 to 0.80, log-rank p = 0.022) **(Fig 2C).** However, after controlling for covariates in a Cox model the association of OS with TS was no longer significant (HR 0.47, 95% CI: 0.31 to 0.94, p = 0.16), replaced by an association between ECOG performance status and survival (HR 0.35, 95% CI: 0.28 to 0.48, p = 0.006). There was a slight trend in the same direction for median TTF in the low TS expression group to the high expression group (12.3 vs.11.7 mo, HR 0.82, 95% CI: 0.33 to 2.0, Log-rank p = 0.18) that did not reach statistical significance. **(Fig 2D).** The ORR to any therapy in the patients with low TS was 86% compared to 58% in the high TS level group (RR 0.68, 95% CI: 0.41 to 1.1, Fisher’s exact p = 0.10) **(Table 2).**

### UCSD Cohort, ERCC1/TS Combination

**Fig 3:**
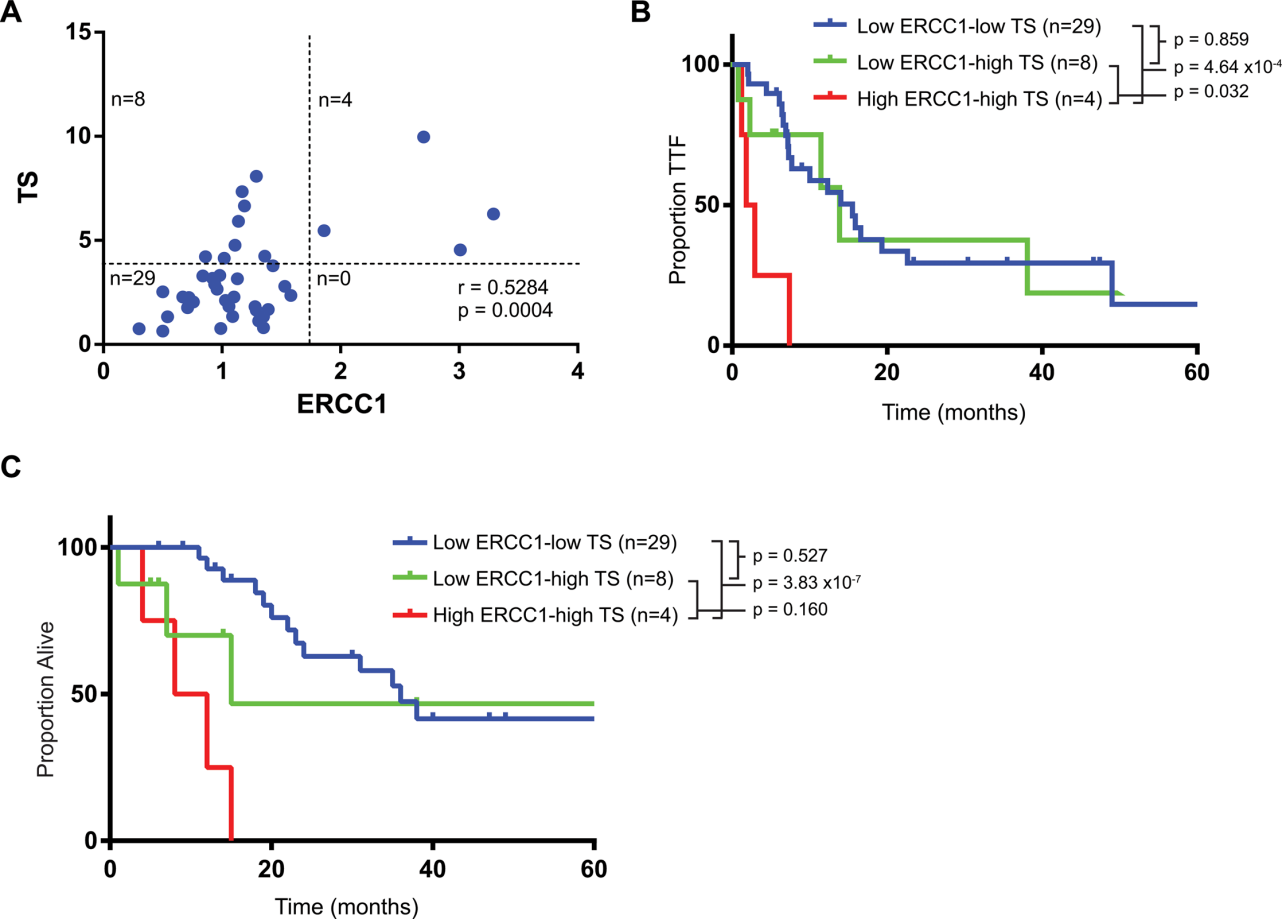
Combination of ERCC1 and TS. (A) Correlation between ERCC1 and TS expression. (B) Time to treatment failure of all patients per combination of ERCC1/TS. (B) Overall survival of all patients per combination of ERCC1/TS

We then evaluated if ERCC1 and TS could be combined to form a composite marker capable of identifying sub-populations with significant differences in TTF, ORR and OS. Twenty-nine patients had low expression levels of both ERCC1 and TS, eight had low expression levels of ERCC1 but elevated TS, and four patients had elevated levels of both ERCC1 and TS. No patient displayed the combination of high ERCC1 with low TS. Interestingly, expression levels of ERCC1 and TS were significantly correlated (Pearson *r* = 0.53; p = 0.0004) **(Fig 3A)**. Patients with high ERCC1 and TS had the shortest TTF of only 2.4 months, compared to 14.1 months in patients with low ERCC1 and high TS and 15.5 months in patients with low levels of both ERCC1 and TS **(Fig 3B)**. The difference in median TTF between the low ERCC1-low TS cohort relative to high ERCC1-high TS (15.5 vs. 2.4 mo, HR 0.17, 95% CI 0.0012 to 0.11, log-rank p = 4.6x10^-4^) was statistically significant and remained significant after control for covariates (HR 0.13, 95% CI: 0.11 to 0.16, p = 0.0029). Similar trends were seen with OS, high ERCC1-high TS patients having the shortest median OS of 10 months compared to 15 months for low ERCC1-high TS and 36 months for low ERCC1-low TS. The difference in median OS between the low ERCC1-low TS cohort relative to high ERCC1-high TS (36 vs. 10 mo, HR 0.10, 95% CI 3.4x10^-5^ to 0.0088, log-rank p = 3.8x10^-7^) was also statistically significant and remained so after control for covariates (HR 0.092, 95% CI: 0.078 to 0.11, p = 0.014).

Patients with an elevated level of both ERCC1 and TS had a trend towards lower ORR of 50% compared to 63% in patients with low ERCC1/high TS and 86% in patients with low levels of both markers (**Table 2**). Patients with low expression of both ERCC1 and TS had a particularly high response rate of 91% when treated with FOLFOX. Amongst all patients treated with FOLFOX those with dual low expression had a higher response rate relative to those with high expression of either or both ERCC1/TS (40% vs. 91%, RR 2.3, 95% CI: 0.77 to 6.7, Fisher’s exact test p = 0.03, n = 27). The combined ERCC1/TS biomarker was not predictive of response to FOLFIRI (71% vs. 71%, RR = 1.0, Fisher’s exact test p = 1, n = 14) **(Table 3)**. All but one of the patients that went on to undergo metastatectomy had low expression of both genes. The rate of metastatectomy in patients with elevated expression of either gene was 8.3% (1/11), compared to 27% (10/27) for those with dual low expression (RR 3.2, 95% CI: .46 to 23, Fisher’s exact p = 0.252).

**Table 3:** Objective response rates per chemotherapy, stratified by biomarker.

treatment	low ERCC1 & low TS	either or both ERCC1 or TS high	p value (Fisher Exact test)
FOLFOX	20/22 (91%)	2/5 (40%)	0.030
FOLFIRI	5/7 (71%)	5/7 (71%)	1

## Discussion

Achieving a clinical response to first line therapy is of critical importance in mCRC as it is known that many of these patients will not be fit enough to tolerate a combination of cytotoxic chemotherapeutic drugs in the second line due to decline in performance status. FOLFOX and FOLFIRI, the two major backbone chemotherapy regimens used to treat mCRC have been shown to have equivalent response rates when averaged across a large cohort of mCRC patients[3]. However, given the tremendous molecular diversity between different individual colon tumors[31], presumably there are subsets of tumors more likely to response to one regimen than the other. However, for patients with metastatic colon cancer, currently the majority of cytotoxic chemotherapy is given without any biomarker to predict response. It was the goal of this study to investigate whether molecular data from the commercially available gene expression and mutation panel ResponseDx Colon could be used to predict response and TTF to first line therapy.

Our study design was inherently limited by its retrospective design and modest sample size, and was further limited by the smaller than expected number of patients with high ERCC1 expression. It should be noted we used previously established cutoffs for ERCC1 and TS[6]. Due to these limitations the study was powered to detect only particularly large magnitudes of effects with statistical significance. Despite these limitations, an observation of potential clinical importance can be made that patients with low levels of both ERCC1 and TS had a remarkably high response rate of 91% (20/22) to FOLFOX. It is difficult to make a direct comparison to the FOLFIRI response rate of 71% (5/7) given the small sample size, however if these response rates are confirmed in larger studies this finding would suggest that in the absence of contraindications to platinum therapy, FOLFOX may be the preferred first line of treatment in these patients. These patients with low ERCC1 and TS expression had a significantly longer TTF relative to patients with high expression for either or both genes regardless of whether they were given first line FOLFOX or FOLFIRI, suggesting dual low expression of ERCC1/TS could be a marker of general chemo-sensitivity rather than specifically platinum sensitivity.

The twelve patients with tumors expressing high levels of either TS or ERCC1 trended towards worse response rate relative to those with dual low expression. The magnitude of difference would be clinically significant if confirmed in larger studies, however due to limited sample size it did not reach statistical significance in this study. Interestingly the response rate to FOLFIRI was the same for tumors with dual low expression and those with high expression of either or both ERCC1 or TS. In contrast, the response rate to FOLFOX was only 40% (2/5) for tumors with high expression of either or both ERCC1 or TS. Although conclusions drawn on a sample of this size should be treated with caution, these results support FOLFIRI as first line chemotherapy for tumors with high expression of either ERCC1 or TS. With similar caution we report the dismal prognosis of the four patients with high expression of both ERCC1 and TS, having TTF of only 2.4 months and OS of 10 months. There were no patients with high ERCC1 and low TS, so the relative contribution of ERCC1 and TS to this observed poor prognosis cannot be assessed in this study. There was only one patient with high ERCC1 and high TS in our cohort who was treated with FOLFIRI, and this patient did not respond, potentially suggesting that the concomitant elevation in ERCC1 and TS expression confers resistance to multiple types of chemotherapy.

We examined the association of ERCC1 and TS expression with OS in TCGA pan-cancer and mCRC cohorts to see if the observed trends would generalize to these independent cohorts. For ERCC1 there was a slight trend toward worse OS with high expression in both pan-cancer and mCRC cohorts, the magnitude of which was exaggerated in patients treated with platinum chemotherapy. This finding is consistent with the known function of ERCC1 in DNA repair following platinum therapy. For TS there was significantly worse OS with high expression in the TCGA pan-cancer cohort, but this effect was not seen in the mCRC cohort.

In conclusion, this study demonstrates the potential utility of ERCC1 and TS gene expression as prognostic and possibly predictive biomarkers in mCRC, consistent with prior studies [6]. It identifies a population with poor prognosis (high ERCC1/TS expression) as well as a population with a remarkably high response rate to FOLFOX chemotherapy (low ERCC1/TS expression). In the future the prognostic and predictive value of ERCC1 and TS expression should be examined the context of a larger prospective clinical trial.

## Supporting Information

S1 Table Patient Characteristics. Table showing demographic, histologic, molecular, and treatment data for the 41 patients included in the UCSD cohort.
(XLSX)
